# Hypoactivation and Dysconnectivity of a Frontostriatal Circuit During Goal-Directed Planning as an Endophenotype for Obsessive-Compulsive Disorder

**DOI:** 10.1016/j.bpsc.2017.05.005

**Published:** 2017-11

**Authors:** Matilde M. Vaghi, Adam Hampshire, Naomi A. Fineberg, Muzaffer Kaser, Annette B. Brühl, Barbara J. Sahakian, Samuel R. Chamberlain, Trevor W. Robbins

**Affiliations:** a Behavioural and Clinical Neuroscience Institute, University of Cambridge, Cambridge, United Kingdom; b Department of Psychology, University of Cambridge, Cambridge, United Kingdom; c Department of Psychiatry, University of Cambridge, Cambridge, United Kingdom; d Cambridge and Peterborough NHS Foundation Trust, Cambridge, United Kingdom; e Cognitive Computational and Clinical Neurosciences Laboratory, Imperial College London, London, United Kingdom; f National Treatment Service for OCD (England and Wales), Hertfordshire, United Kingdom; g Department of Psychiatry, Psychotherapy and Psychosomatics, University Hospital of Psychiatry, Zurich, Switzerland

**Keywords:** Connectivity, Endophenotype, Frontostriatal circuits, Goal-directed, Obsessive-compulsive disorder, Planning

## Abstract

**Background:**

The symptoms of obsessive-compulsive disorder (OCD) have been postulated to result from impaired executive functioning and excessive habit formation at the expense of goal-directed control and have been objectively demonstrated using neuropsychological tests in such patients. This study tested whether there is functional hypoactivation as well as dysconnectivity of discrete frontostriatal pathways during goal-directed planning in patients with OCD and in their unaffected first-degree relatives.

**Methods:**

In total, 21 comorbidity-free patients with OCD, 19 clinically asymptomatic first-degree relatives of these patients, and 20 control participants were tested on a functional magnetic resonance optimized version of the Tower of London task. Group differences in brain activation during goal-directed planning were measured together with associated frontostriatal functional connectivity.

**Results:**

Patients with OCD and their clinically asymptomatic relatives manifested hypoactivation of the right dorsolateral prefrontal cortex during goal-directed planning coupled with reduced functional connectivity between this cortical region and the basal ganglia (putamen).

**Conclusions:**

Hypoactivation of cortical regions associated with goal-directed planning and associated frontostriatal dysconnectivity represent a candidate endophenotype for OCD. These findings accord with abnormalities in neural networks supporting the balance between goal-directed and habitual behavior, with implications for recent neuropsychological theories of OCD and the major neurobiological model for this disorder.

SEE COMMENTARY ON PAGE 638

The major neurobiological model for obsessive-compulsive disorder (OCD) implies functional abnormalities within frontostriatal circuits (1). These circuits are crucial for enabling the successful implementation of flexible, goal-directed behavior (2). Accordingly, deficits in executive functions have been demonstrated in OCD [for a review, see (3)]. In addition, exaggerated appetitive (4) and aversive (5) habit learning in OCD has been shown, also compatible with abnormal activity of corticostriatal circuits affecting the balance between goal-directed and habitual behavior 6, 7. This neuropsychological signature is congruent with the clinical phenotype of patients with OCD, characterized by recurrent intrusive thoughts (obsessions) and/or repetitive behaviors (compulsions) performed at the expense of goal-directed purposeful actions.

In this study, we tested whether abnormalities of the goal-directed system are directly related to state (i.e., symptoms or confounds such as chronic disease and treatments) or whether they are state independent (manifest in an individual whether or not the illness is active) (8). To this end, we used a familial study design that recruited not only patients with OCD but also their first-degree unaffected relatives together with healthy comparison subjects without a family history of the condition. OCD runs in families, with first-degree relatives showing increased risk for the disorder compared with the normal population. There is strong evidence that OCD has a genetic basis, accounting for 40% of the phenotypic variance according to twin studies (9). However, a lack of clear genetic findings has led to the search for candidate endophenotypes, here operationalized as behavioral, cognitive, or neural markers of the disorder detected in clinically unaffected first-degree relatives of patients (8). These endophenotype markers represent stable phenotypes that might reflect a genetic effect and be more closely related to the causes of a disorder than to its symptoms (8).

To investigate the neural basis of the goal-directed system, we used functional magnetic resonance imaging in conjunction with a well-validated goal-directed planning task testing the ability to select the correct sequence of subgoals by anticipating the consequences of one course of action for another. Our investigation was based on substantial overlap between the neural circuitry typically recruited during the execution of goal-directed planning and the frontostriatal circuitry implicated in OCD according to the prevailing neurobiological model (1).

Accordingly, there is evidence of impaired performance with lengthened response times 10, 11, 12 and impaired accuracy under more difficult task conditions 13, 14 in patients with OCD. Such impaired performance persisted despite successful pharmacological treatment (15) and was also found in unaffected relatives of patients with OCD compared with healthy subjects (16), highlighting planning performance as a potential endophenotype for OCD. In spite of a few negative findings 11, 12, several meta-analyses have provided evidence for a considerable impairment in planning abilities in patients with OCD 17, 18. Such impairment was independent from depression, medication, and symptom severity (18) and was evident when analyzing accuracy and response times either as a composite measure or separately. Here, we included only the easy trials to avoid brain activation’s being confounded by differences in performance. Therefore, we did not expect reduced accuracy in patients, in line with previous studies for which such impairment was found only at the most difficult levels of the task (3). Because previous studies have found evidence of lengthened response times 10, 11, 12, we expected increased response times in patients and hypothesized that such abnormalities would extend to their relatives as well.

At the neural level, hypoactivity of the dorsolateral prefrontal cortex (DLPFC) and striatal regions has been identified during goal-directed planning in patients with OCD (19), and high levels of obsessive-compulsive symptoms were associated with hypoactivation of the DLPFC during planning 20, 21. To date, no studies have investigated the neural correlates of goal-directed planning in unaffected first-degree relatives of OCD, which were tested here with the hypothesis of decreased responsiveness in both patients and their relatives.

Moreover, extending the frontostriatal model for OCD (1), dysfunctional interactions between cortical and subcortical nodes, rather than damage to individual brain regions, have been hypothesized as a potential determinant of OCD (22). Recent research has documented a double dissociation for specific frontostriatal circuits, whereby weakened caudate–ventrolateral PFC and putamen–DLPFC resting state connectivity accounted for impaired cognitive flexibility and goal-directed planning in patients with OCD, respectively (14). Here, we probed task-dependent online functional connectivity (FC) during goal-directed planning and tested the a priori hypothesis of reduced frontostriatal connectivity between frontal and subcortical brain regions during the implementation of goal-directed sequences in OCD patients. In addition, leveraging a familial study design, we tested whether reduced frontostriatal connectivity represented a candidate endophenotype for OCD.

## Methods and Materials

### Participants

In total, 21 comorbidity-free patients with OCD, 19 of their clinically unaffected first-degree relatives, and 22 control subjects participated in the study. Groups were matched for age and verbal IQ. All participants were right handed, and each group included a similar proportion of female participants (see Supplement). Patients reported higher levels of depressive symptoms as assessed with the Montgomery–Åsberg Depression Rating Scale (23), albeit well below the clinical threshold (Table 1), and the majority of these (*n* = 17; Supplement) were receiving selective serotonin reuptake inhibitors.

**Table 1:** Demographic, Clinical, and Behavioral Characteristics of Patients With OCD, Their First-Degree Unaffected Relatives, and Healthy Comparison Subjects

	Control Subjects (*n* = 20)	Relatives (*n* = 19)	Patients (*n* = 21)	Statistic	*df*	*p*
Demographic Measures						
Gender, *n* (male:female)	5:15	5:14	3:18	χ^2^ = 1.047	2	.592
Age, years	36.45 (8.54)	41.11 (10.63)	37.90 (14.31)	*F* = 0.835	2,57	.439
Estimated verbal IQ	115.80 (6.07)	114.57 (7.04)	115.55 (5.13)	*F* = 0.220	2,57	.803
Handedness	61.50 (48.15)	61.58 (44.38)	65.71 (46.86)	*F* = 0.055	2,57	.947
Clinical Measures						
MADRS	1.35 (3.38)	2.32 (3.20)	7.33 (7.34)	*F* = 8.178	2,57	.001
Y-BOCS obsessions	0	0	11.43 (2.82)			
Y-BOCS compulsions	0	0	12.05 (2.71)			
Y-BOCS total	0	0	23.00 (5.65)			
Behavioral Measures						
Planning accuracy, overall %	85.36 (3.15)	82.01 (3.23)	84.68 (3.07)	*F* = 0.623	2,57	.540^a^
Accuracy, p2 %	88.69 (15.98)	85.66 (19.89)	87.86 (11.19)			
Accuracy, p3 %	87.74 (14.15)	83.09 (18.27)	85.47 (17.65)			
Accuracy, p4 %	79.64 (21.92)	77.28 (21.82)	80.71 (21.88)			
Planning response times, overall seconds	6.58 (0.66)	8.47 (0.68)	8.81 (0.65)	*F* = 4.236	2,57	.019^b^
Response time, p2 seconds	5.97 (1.96)	7.25 (2.05)	6.96 (2.05)			
Response time, p3 seconds	5.98 (1.51)	8.52 (2.79)	8.47 (4.93)			
Response time, p4 seconds	7.79 (2.64)	9.64 (2.85)	11.00 (6.29)			

Values are presented as mean (SD). Estimated verbal IQ was measured with the National Adult Reading Test. Handedness was measured with the Edinburgh Handedness Inventory.

MADRS, Montgomery–Åsberg Depression Rating Scale; OCD, obsessive-compulsive disorder; p2, planning 2 moves; p3, planning 3 moves; p4, planning 4 moves; Y-BOCS, Yale-Brown Obsessive Compulsive Scale.

a Repeated-measures analysis of variance; there was not a main effect of group for accuracy.

b Repeated-measures analysis of variance; there was a main effect of group on response times, with patients and relatives being slower than controls irrespective of trial type (counting or planning) and difficulty level (2, 3, or 4 moves).

### Experimental Design

**Figure 1:**
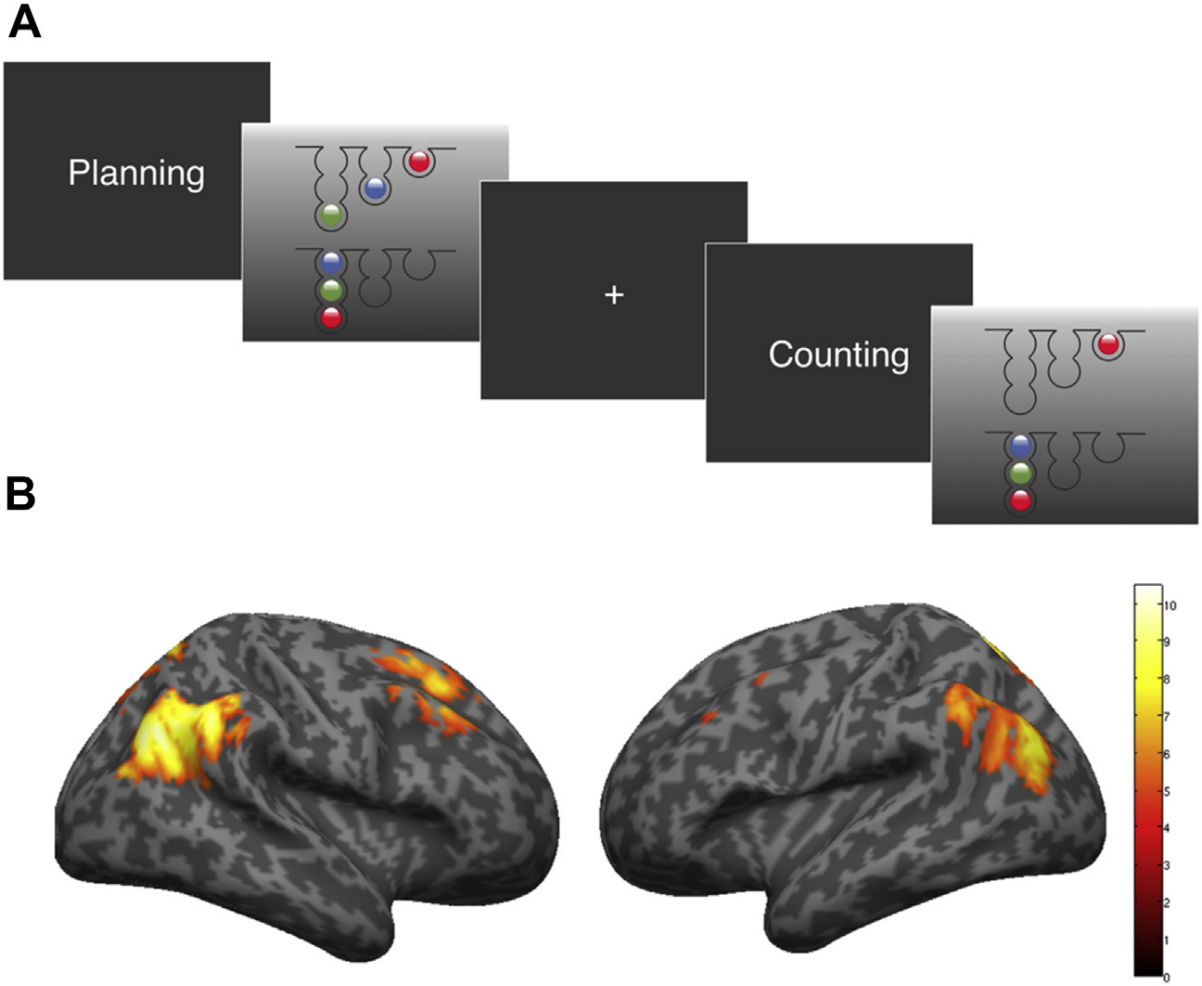
Experimental design and associated brain network for goal-directed planning. **(A)** Display screen of the One-Touch Spatial Planning task used in the current study. Participants were presented with a cue screen displaying the word “Planning” (planning trials) or “Counting” (counting trials) before each new problem to disambiguate between trials. In the planning condition, participants indicated by pressing the corresponding button on a functional magnetic resonance imaging compatible button box the minimum possible number of moves needed to rearrange the balls in the upper half of the screen so that they were in the same configuration as those in the lower half of the screen. Participants were instructed that the balls needed to move in and out of the top of the tubes, could be moved only one at a time, and could not be moved past each other in the tubes. In the counting condition, participants needed to subtract the number of balls in the top array from the number of balls in the bottom array. Feedback for correct and incorrect answers was provided at the end of each trial. Difficulty varied from 2 to 4 moves. Event duration was measured from the time of appearance of the stimulus until the time of a response on the button box. **(B)** Main effect of planning (planning minus counting) across all participants (*n* = 60). Voxelwise familywise error correction for the whole-brain volume; *p* < .05. Significant brain activation was identified in the expected frontoparietal network during planning.

We used the One-Touch Spatial Planning task (24) to test goal-directed planning and the ability to achieve a goal through intermediate steps (Figure 1A). The task represents a functional magnetic resonance imaging variant of the corresponding Cambridge Neuropsychological Test Automated Battery paradigm (Supplement; see imaging parameters and preprocessing), whereby in addition to planning trials, a counting condition controls for visual, attentional, and motor demands. Problems with 2, 3, or 4 correct moves were included. Planning and counting problems were displayed alternately. The experimental paradigm lasted for approximately 10 minutes (Supplement).

### Data Analysis

#### Behavior

Behavioral data were analyzed with a standard statistical package (SPSS version 23.0, IBM Corp., Armonk, NY). Behavioral performance was assessed by the percentage of problems attempted that were responded to correctly (accuracy) and the associated mean response times. For each of these measures, a 2 × 3 × 3 repeated-measures analysis of variance (ANOVA) compared performance between groups. The within-subject factors were trial type (planning or counting) and difficulty level (2, 3, or 4), and the between-subject factor was group (controls, relatives, or patients). Appropriate corrections were applied in case of violation of the assumption of sphericity. When significant differences were found with ANOVA, post hoc protected least significant difference tests were conducted. The relationship between behavioral measures (accuracy and mean response times) and disease severity (Yale-Brown Obsessive Compulsive Scale [Y-BOCS]) was investigated in patients with OCD.

#### Functional Magnetic Resonance Imaging

For imaging data analysis, SPM was used. The hemodynamic response was modeled to the onsets and durations of planning and counting problems. Onsets were the time of appearance of the stimuli on the screen, and durations were measured to the time of response. For each individual, the following contrasts of interest were estimated: planning activation relative to rest for 2, 3, and 4 moves; planning activation relative to counting for 2, 3, and 4 moves, respectively; planning high minus planning low; planning minus counting; and counting minus planning (Supplement). Whole-brain maps depicting these contrast estimates were collated for second-level (group) random-effects analyses as specified in the Supplement for each contrast. Activation was deemed significant at *p* < .05, familywise error (FWE) corrected at the voxel level, or *p* < .001 uncorrected.

Previous studies have demonstrated caudate activation in association with planning 25, 26, 27 and reduced activation in caudate and putamen during planning in patients with OCD (19). Thus, regions of interest (ROIs) in those regions were defined in both hemispheres as 3.5-mm radial spheres using the MarsBaR toolbox at Montreal Neurological Institute (MNI) coordinates *x* = 11, *y* = 7, *z* = 9 for the caudate and at MNI coordinates *x* = 24, *y* = 0, *z* = 3 for the putamen (Supplement). Average intensity value from all voxels within an ROI was calculated to carry out ROI analyses. Neural responses were correlated with behavioral measures and, to explore their relationship with symptom severity, with Y-BOCS values and depression scores.

#### Functional Connectivity

Because caudate and putamen were hypothesized to mediate executive performance via task-related FC modulation with the PFC 2, 28, we conducted a psychophysiological interaction (PPI) analysis to interrogate frontostriatal connectivity during goal-directed planning. In accordance with previous research with this paradigm (24), an ROI was derived from the main effect of planning minus counting across all subjects thresholded at *p* < .05, FWE corrected, and corresponding to the peak coordinate of the cluster including the right superior and middle frontal gyri (MNI coordinates *x* = 24, *y* = 20, *z* = 52). PPI analysis was conducted as described in the Supplement. We generated our hypothesis of reduced frontostriatal FC in patients with OCD and their first-degree unaffected relatives on the basis of previous findings showing DLPFC hypoactivation in patients with OCD during goal-directed planning (19) and on findings of reduced resting-state connectivity between the DLPFC and the putamen accounting for impoverished performance on goal-directed planning in patients with OCD (14). Therefore, we expected hypoactivation of DLPFC to be reflected in reduced interaction with associated striatal structures during goal-directed planning, both in patients with OCD and in their first-degree relatives, as tested by nonparametric permutation testing (one tailed, Monte Carlo pairwise permutation testing conducted in SPSS; see Supplement). Significance was set at *p* < .05. FC parameters were correlated with behavioral measures and with Y-BOCS and depression values to investigate the relationship between symptoms and frontostriatal connectivity.

## Results

### Behavioral Results

Accuracy did not differ across groups (*F*_2,57_ = 0.623, *p* = .540). All participants achieved a lower number of correct responses during planning trials versus counting trials (*F*_1,57_ = 23.033, *p* < .001). The proportion of correct responses decreased with problem difficulty (*F*_2,57_ = 9.377, *p* < .001), with no significant trial type and difficulty interaction (*F*_1.87,106.48_ = 0.430, *p* = .638). There was no significant interaction between group and trial type (*F*_2,57_ = 0.632, *p* = .40) or between group and difficulty (*F*_4,114_ = 0.380, *p* = .822) (Supplemental Figure S1A, B and Table 1). Repeated-measures ANOVA revealed a main effect of group for response times (*F*_2,57_ = 4.236, *p* = .019), with patients (*p* = .009) and relatives (*p* = .027) being slower than control subjects irrespective of trial type and difficulty. Across all groups, response times were significantly longer in the planning condition (*F*_1,57_ = 348.897, *p* < .001). A main effect of difficulty (*F*_1.63,93.06_ = 24.178, *p* < .001) indexed significantly prolonged response times across trial type and groups for the most difficult problems. The interaction between trial type and difficulty (*F*_1.98,112.84_ = 26.627, *p* < .001) indicated that response times during planning were significantly lengthened as the difficulty of the problems increased across groups. There was no significant interaction between group and trial type (*F*_2,57_ = 0.270, *p* = .765) or between group and difficulty (*F*_4,114_ = 1.462, *p* = .218) (Supplemental Figure S1C, D). There were no significant correlations between Y-BOCS scores and accuracy (all *p*s > .286) or reaction times (all *p*s > .057) in patients with OCD. In summary, both patients and relatives had intact accuracy but slower responding compared with control subjects.

### Brain Regions Activated During Goal-Directed Planning

#### Planning Minus Counting

A robust effect of planning minus counting was found in the expected dorsal frontoparietal network across all participants (*p* < .05, FWE) (Figure 1B and Supplemental Table S1). This network was different from the one identified on the opposite contrast, counting minus planning (Supplemental Figure S2 and Supplemental Table S2).

Although we did not observe significant striatal activation in association with the planning component, ROI analysis was conducted for the right putamen and right caudate for the contrast planning minus counting. Parameter estimate signal change was explored separately in each group by means of one-sample *t* test. Significant deactivation of the right putamen was found in control subjects (*t*_19_ = −4.76, *p* < .001) and in relatives of patients with OCD (*t*_18_ = −2.479, *p* = .023), but not in patients (*t*_20_ = −0.837, *p* = .412). This analysis indicated insufficient hypoactivation of the putamen during planning in patients with OCD, as confirmed by significant differences in patients compared with control subjects (*U* = 109.000, *Z* = −2.634, *p* = .007, Monte Carlo pairwise permutation testing, Bonferroni corrected, SPSS). There was no difference between relatives and control subjects (*U* = 139.000, *Z* = −1.433, *p* = .158) (Supplemental Figure S3A). No significant involvement of the right caudate was found in control subjects (*t*_19_ = −0.152, *p* = 0.880), relatives of patients with OCD (*t*_18_ = 0.311, *p* = .760), or patients (*t*_20_ = 1.549, *p* = .137) (Supplemental Figure S3B) (but see the Supplement for evidence of caudate involvement on the contrast planning minus resting).

#### Between-Group Effects on Goal-Directed Planning

**Figure 2:**
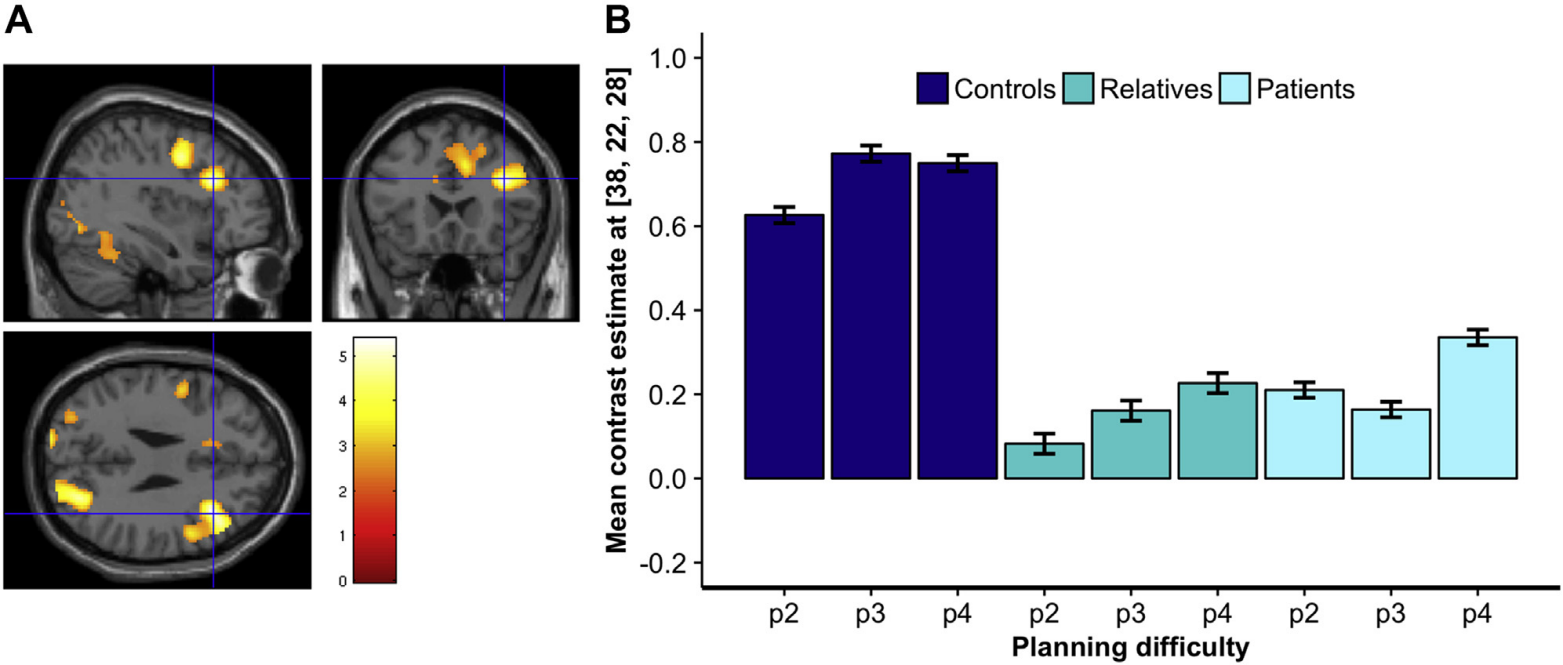
Brain areas of hypoactivation in patients with obsessive-compulsive disorder and their first-degree unaffected relatives during goal-directed planning. **(A)** At all levels of planning complexity, patients with obsessive-compulsive disorder and their first-degree relatives showed hypoactivation in an extensive cluster in the right middle frontal gyrus, including Brodmann areas 6, 9, and 9/46. Brain activation rendered at *p* < .01 corrected for false discovery rate, with 4592 voxels extent threshold for display purposes. **(B)** Parameter estimate in arbitrary units in the dorsolateral prefrontal cortex at peak coordinates *x* = 38, *y* = 22, *z* = 28 (significant at *p* < .05; familywise error, corrected for the whole-brain mass). Error bars denote SEM. p2, planning 2 moves; p3, planning 3 moves; p4, planning 4 moves.

During goal-directed planning, we detected a main effect of group on neural activation in the right precentral gyrus (Brodmann area 6 [BA 6]) and in the middle frontal gyrus entailing BA 9 and BA 9/46 (Table 2) (Supplement; planning minus resting, between-group differences). Post hoc comparisons revealed significant frontal hypoactivation in patients and in their unaffected first-degree relatives compared with control subjects with no evidence of a significant difference between relatives and patients (Table 2). In fact, at all levels of planning complexity, the DLPFC (BA 9 and BA 9/46) was hypoactive in patients and their relatives when compared with control subjects (Table 2 and Figure 2). Covariation for depression scores or response times did not significantly affect the results. Similarly, there was no significant correlation between brain activity at the different levels of planning complexity and response times or accuracy (all *p*s > .360) either when collapsing all participants together or for each group separately. There was no significant correlation with Y-BOCS and depression scores (all *p*s > .331). Similar frontal hypoactivation was identified in patients and relatives (MNI coordinates *x* = 38, *y* = 22, *z* = 26; cluster size = 13, *Z* = 4.39, *p* = .027, FWE) even when analyzing whole-brain between-group differences via a one-way ANOVA on the contrast planning versus resting. There were no differences between patients and relatives.

**Table 2:** Group Differences in Brain Activation During Goal-Directed Planning

Contrast	Region		BA	MNI Coordinates			*k*_*E*_	*Z*	Peak *p*_FWE_
				*x*	*y*	*z*			
Main Effect of Group									
	R	Precentral gyrus	6	44	2	44	16	4.67	.012
	R	Middle frontal gyrus	9	38	22	28	8	4.51	.023
	R	Middle frontal gyrus	9/46	46	28	30	1	4.36	.041
Post Hoc Comparisons									
Controls > patients	R	Middle frontal gyrus	9/46	46	28	30	10	4.51	.019
	R	Middle frontal gyrus	6/9	44	2	44	10	4.58	.021
Controls > relatives	R	Middle frontal gyrus	6/9	36	22	26	14	4.56	.015
	R	Precuneus	19	28	−72	26	18	4.52	.018
	R	Middle frontal gyrus	6/9	42	0	44	12	4.47	.022
	R	Middle frontal gyrus	9	30	0	48	7	4.39	.044
Relatives > patients	–	–	–	–	–	–	–	–	–
Patients > relatives	–	–	–	–	–	–	–	–	–
Controls > both	R	Middle frontal gyrus	6	44	2	44	86	5.16	.001
	R	Middle frontal gyrus	9	38	22	28	93	5.01	.002
	R	Middle frontal gyrus	9/46	46	28	30		4.79	.006
	R	Precuneus		28	−72	28	27	4.56	.015
	R	Middle occipital gyrus	19/18	30	−82	8	1	4.33	.037

Coordinates in MNI space. *p*_FWE_ = *p* value with familywise error correction for the whole-brain volume (*p* < .05).

BA, Brodmann area; FWE, familywise error; *k*_*E*_, cluster size; MNI, Montreal Neurological Institute; R, right; *Z*, *Z* score.

To investigate between-group differences for processes exclusively involved in planning, we compared brain activation across groups on planning versus counting (Supplement; planning minus counting, between-group differences). Hypoactivation was identified in patients and relatives compared with control subjects in the right middle frontal gyrus (BA 6/8) (MNI coordinates *x* = 32, *y* = 8, *z* = 48; cluster size = 14, *Z* = 3.32, *p* < .001, uncorrected). There were no differences between relatives and patients. Covariation of depression scores or response times did not significantly affect the results, and there was no correlation with clinical or behavioral scores. These results suggest that hypoactivation of the DLPFC represents a trait marker of the disorder. To investigate the effect of planning load, we compared groups on high difficulty minus low difficulty of planning (Supplement). Finally, there were no between-group differences for counting trials (Supplement).

#### Frontostriatal Connectivity During Goal-Directed Planning

In control subjects, significant frontal connectivity was found with the putamen (*t*_18_ = 5.931, *p* < .001, Bonferroni corrected for multiple comparisons) but not with the caudate (*t*_18_ = 1.866, *p* = .078). This suggested frontoputaminal connectivity to be implicated during planning and therefore was further explored in patients and relatives. While strong PPI connectivity between the frontal seed and the putamen was observed in control subjects, there was no such significant connectivity in relatives (*t*_17_ = 0.738, *p* = .471) or in patients (*t*_20_ = 0.922, *p* = .367).

**Figure 3:**
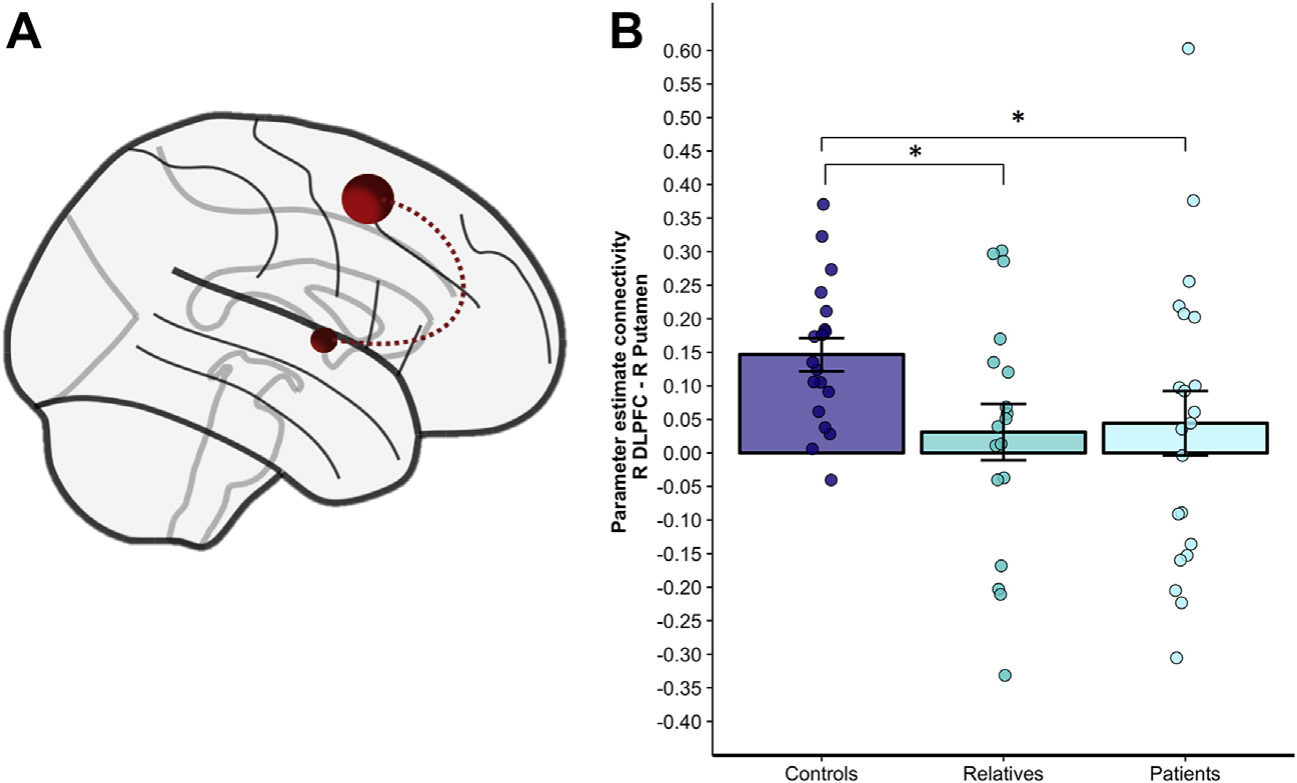
Reduced frontostriatal coupling in patients with obsessive-compulsive disorder and their first-degree unaffected relatives during goal-directed planning. **(A)** Schematic rendering of the seed region for psychophysiological interaction analysis in the right superior frontal gyrus (Brodmann area 6) derived from the main effect of planning minus counting across all subjects (spherical region of interest at *x* = 24, *y* = 20, *z* = 52). Significant group differences were tested for connectivity between the right superior frontal gyrus and the right putamen (spherical region of interest at *x* = 24, *y* = 0, *z* = 3). **(B)** Bar plot showing mean parameter estimate in psychophysiological connectivity in arbitrary units when addressing functional connectivity during goal-directed planning between the right dorsolateral prefrontal cortex (R DLPFC) and the right putamen (R putamen). Compared with control subjects, patients with obsessive-compulsive disorder (*p* = .025, one-tailed Monte Carlo pairwise permutation testing) and first-degree relatives (*p* = .019, one-tailed Monte Carlo pairwise permutation testing) showed reduced functional coupling between the R DLPFC and the R putamen during goal-directed planning. Error bars show SEM. ∗*p* < .05, Monte Carlo pairwise permutation testing, Bonferroni corrected.

When comparing cross-group differences in the frontoputaminal PPI parameter estimates (Figure 3A), patients with OCD showed reduced FC between the seed region and right putamen compared with control subjects (*U* = 127.000, *Z* = −1.964, *p* = .025; Monte Carlo pairwise permutation testing, Bonferroni corrected, SPSS). Similarly, reduced frontoputaminal connectivity was found in relatives when compared with healthy volunteers (*U* = 102.000, *Z* = −2.097, *p* = .019, Monte Carlo pairwise permutation testing, Bonferroni corrected, SPSS) (Figure 3B). PPI FC between the right DLPFC and the right putamen did not correlate with Y-BOCS scores or depression scores. Neither for all participants collapsed together nor for each group separately was there a significant association between brain connectivity and response times or accuracy (all *p*s > .252).

For both the right putamen and right caudate, significant beta weights were found for the physiological predictor for each group separately (all *p*s < .001), and there were no differences across groups.

## Discussion

This study found hypoactivation of the DLPFC during goal-directed planning, not only in patients with OCD but also in their unaffected first-degree relatives when compared with control subjects without a family history of OCD. In addition, the profile of task-dependent frontostriatal interaction during goal-directed behavior was reduced in patients and was indistinguishable from that of their relatives. Therefore, goal-directed planning hypoactivation of the DLPFC and associated frontostriatal connectivity were identified as a hitherto undiscovered candidate endophenotype for OCD.

Reduced activation of the DLPFC in patients with OCD during goal-directed planning is highly consistent with previous findings (19). Here, we independently replicated those findings and provided new evidence that the same pattern of hypoactivation in the DLPFC characterizes patients with OCD and their relatives alike, suggesting a common weak frontal neural recruitment during goal-directed planning. Because patients with OCD and their relatives were matched on all demographic variables, this difference might reflect a genetic or familial susceptibility to OCD. This also rules out the potential confounding effects of medication and symptom duration, indicating that the observed pattern of hypoactivation is a trait marker of OCD. Further pointing to the state-independent nature of these functional abnormalities, as a necessary criterion for an endophenotype (8), there was no association between the neuroimaging findings and illness severity in patients.

Frontal and parietal anomalies have been previously shown in patients with OCD and their first-degree relatives. There is evidence of shared structural abnormalities 29, 30 and, at the functional level, hypoactivation related to reversal learning (31) and hyperactivation during response inhibition (32) and working memory performance (33). Together with these studies, our results suggest that endophenotype abnormalities extend across multiple executive domains distributed in specific frontal regions. Our results further demonstrate that while OCD pathophysiology is traditionally linked to the orbitofrontal circuit (6), it is likely associated with a more distributed network (34).

All groups showed significant activation within the caudate during goal-directed planning; in addition, relative to counting, there was a prominent hypoactivation of the putamen in healthy volunteers that was not observed in patients. This finding points to a relative lack of putaminal hypoactivation, an area usually associated with habitual behavior, during a goal-directed task in patients with OCD. We hypothesize that enhanced functioning of the putamen might be linked to an exaggerated habitual tendency observed in OCD 4, 5.

We also observed reduced functional coupling between the DLPFC and the putamen during goal-directed planning in patients with OCD compared with control subjects and, consistent with an endophenotype pattern, in relatives of patients compared with control subjects. Evidence of significant interactions between the DLPFC and putamen is provided by studies in a healthy population showing dense structural interconnections (35) and functional cross-talk as measured during task (28) and at rest (36).

Parallel frontostriatal circuits (2) and nonreciprocal connections (37) between the PFC and the basal ganglia are pivotal for enabling learning and flexibility. These cognitive domains are frequently impaired in OCD, as shown by studies demonstrating deficits in response inhibition 29, 38, reversal learning (31), and extradimensional set shifting (39).

In a sample of patients with OCD different from those used in this study, a direct relationship was recently demonstrated between reduced DLPFC–putamen resting-state connectivity and impaired performance in goal-directed planning tested outside the scanner (14). Here, we have extended those findings by showing, remarkably, that exactly the same pattern of neural dysfunctional interaction can be discerned during the implementation of goal-directed planning and that it might represent a neurocognitive trait for the disorder.

A limitation of our analysis is that it does not allow us to draw conclusions on directionality; however, a plausible mechanism might be a failure of top-down control over subcortical brain regions. Overall, insufficient DLPFC top-down recruitment in patients and relatives might result in weak frontostriatal interaction and lack of top-down control of the putamen, potentially contributing to impairments in the ability of assembling novel sequences of behavior over potentially disruptive habitual tendencies inherent in the task. Thus, this may contribute to the inability of patients with OCD to suppress automatic behavior in favor of flexible, goal-oriented behavior.

Frontostriatal abnormalities are also found on tasks assessing implicit learning 40, 41, 42. The task used here allowed only the investigation of the goal-directed system; however, further studies are needed to understand the relationship between the goal-directed system and systems allocated for skill learning implicating, for example, the cerebellum.

The behavioral data showed longer response times in patients with OCD and their relatives, whereas accuracy scores were not significantly affected. A potential limitation is that the measure of accuracy was based only on the predicted number of moves required to solve the problems. Even if participants took longer to solve more complicated problems, they might have simply ruled out that solutions with lower numbers of moves were not possible. However, our findings agree with previous studies identifying significantly reduced accuracy only at the most difficult levels of planning, which were intentionally not included in this study. The most parsimonious interpretation of our results is that the same level of accuracy in patients and relatives as in control subjects was obtained at the cost of their lengthened response times. Alternatively, because the degree of certainty that a decision is correct is also indexed by the decision time 43, 44, it is possible that longer response times reflected increased uncertainty. Slowing was found not only on planning trials but also on counting trials. However, between-group differences for goal-directed planning were not due to different response times, and there were no significant group differences in the counting condition. It is striking that the same pattern was found not only in patients but also in their first-degree unaffected relatives. Our main approach, consistent with earlier studies (45), did not make use of a control condition that included all visuomotor and cognitive processes other than those implicated specifically in planning. An obvious limitation of this approach is that, although it captures primarily planning-related activation, it may also tap into secondary nonspecific aspects of attention and visuospatial perception. However, even when using a suitable contrast (i.e., counting), we obtained similar results for between-group differences, albeit at a lower statistical level of significance. Moreover, a more stringent contrast was used in the FC analysis, thereby isolating planning components such as working memory and response sequencing in goal-directed behavior. Brain imaging activation did not differ for counting trials across groups, indicating no obvious group differences for those processes merely involved in the counting task, including visuospatial attention.

In keeping with previous imaging studies, the frontoparietal network was found to be selectively activated during planning minus the attentional and visuomotor control condition (25). Lateralization toward the right side is consistent with previous studies emphasizing the role of the right hemisphere when the task requires manipulation of task-related information (46) rather than reproduction of the appropriate motor sequence for which the left hemisphere is more heavily involved and minimized by our experimental design 25, 47.

Similar to previous studies 24, 25, 26, 45, we did not detect caudate activation when planning trials were compared with a control condition. However, modulation of subcortical brain regions was found in association with planning load in control subjects, also consistent with previous findings 25, 26.

The fact that most patients were medicated with selective serotonin reuptake inhibitors, which could affect connectivity measures in OCD (48), poses a potential limitation for this study. We did not have sufficient power to make a direct comparison between medicated and unmedicated patients. Similarities between patients and completely unmedicated relatives of patients with OCD, however, militate against the notion that findings were significantly mediated by a medication effect. Nevertheless, in subsequent studies, an appropriate unmedicated group of patients with OCD should be included to definitively determine the effect of medication.

Finally, although we carefully excluded any participants with anxiety disorders and any other comorbidity, we did not record subclinical anxiety scores. Previous studies tested brain correlates of planning performance transdiagnostically, revealing only subtle differences between patients with OCD and patients affected by panic disorder or hypochondriasis and suggesting that frontostriatal and limbic abnormalities during planning might be independently implicated across these disorders (49). A key question not directly investigated here therefore pertains to whether the observed hypoactivation is specific to OCD. Because the same pattern of hypoactivation is found in unaffected first-degree relatives, our results rule out the hypothesis that state components (i.e., symptoms or confounds such as chronic disease and treatments) directly contribute to the observed neural readout.

Even if endophenotypic traits might be relatively less complex genetically than disease phenotypes, it is nevertheless plausible that they may reflect the operation of many different genes. Endophenotypic traits might also predate signs or symptoms of illness. However, the first-degree unaffected relatives included in this study were beyond the typical age of onset for OCD. Therefore, even if the identified neural and behavioral markers might predispose to the development of OCD, they do not seem sufficient in themselves to lead to the full-blown manifestation of the condition. It remains to be understood how this familial vulnerability trait, which could also result from environmental factors such as stress, results in OCD symptoms.

In conclusion, we have identified hypoactivation in the DLPFC as a candidate neurocognitive endophenotype for OCD. Associated aberrant coupling with the putamen might predispose toward excessive habit formation and suboptimal goal-directed performance.
