# Supplementary material for: Hypoactivation and Dysconnectivity of a Frontostriatal Circuit During Goal-Directed Planning as an Endophenotype for Obsessive-Compulsive Disorder
Source: Biol Psychiatry Cogn Neurosci Neuroimaging. 2017 Nov;2(8):655–63. doi: 10.1016/j.bpsc.2017.05.005 (PMC5684958; doi:10.1016/j.bpsc.2017.05.005)
Supplement: Supplemental Material [file mmc1.pdf]

Hypoactivation and Dysconnectivity of a Frontostriatal Circuit During Goal-directed Planning as an Endophenotype for Obsessive-Compulsive Disorder

Supplementary Information

SUPPLEMENTAL METHODS

Participants

Control participants were recruited from the community, they were not taking psychiatric medication and had never suffered from a psychiatric condition. In addition, controls were not enrolled in the study in case their first-degree relatives were affected by psychiatric conditions. However, one control participant revealed only at the time of testing that he had a son with autistic spectrum disorder and was thus excluded for further analyses. One control participant was excluded due to structural abnormalities. Patients were screened by a psychiatrist to ensure they met the criteria for OCD and had no comorbid psychiatric disorders or exhibition of depressive episodes. In line with many other studies from our group, patients were enrolled in the study only if their symptom severity exceeded a threshold of 12 on the Yale-Brown Obsessive Compulsive Scale (Y-BOCS) [1] therefore excluding patients with subclinical OCD (Y-BOCS scores between 0 and 7). The majority of the included patients either qualified for moderate (Y-BOCS scores between 16 and 23, n=11), severe (Y-BOCS scores between 24 and 31, n=6) or extreme (Y-BOCS scores between 32 and 40, n=3) symptomatology. Only one patient, above our clinical threshold, qualified for mild OCD (Y-BOCS scores between 8 and 15). Patients for whom hoarding was the primary complaint were excluded. Seventeen patients were medicated, receiving stable doses of serotonin reuptake inhibitors (SSRIs), 4 patients were unmedicated at the time of the study. We recruited first-degree unaffected relatives of the OCD patients who were included in the study. First-degree relatives were unmedicated and never suffered from a psychiatric

disorder. They were either brother or sister of the proband, generally of the same sex unless this was not possible due to psychiatric illness or unavailability. This strategy also allowed careful matching for age.

All participants reported no history of head trauma, neurological disease, or contraindications for MRI. No left handed participants were included in the study as assessed via the Edinburgh Handedness Inventory [2]. Data for many of these subjects were reported previously in relation to a different cognitive task [3] and only clinical scales measuring depression and OCD symptoms were administered to the participants.

This study was approved by the local research ethics committee and all participants gave written informed consent prior to participation.

Experimental Design

A modified version of the Tower of London [4] was chosen as experimental task because it is known to reliably activate prefrontal association cortex in both positron emission tomography [5–8] and functional MRI studies (fMRI) [8–11] and it has been well validated as a measure of executive dysfunction in OCD [12].

In the task employed, on a given trial, the participant was presented with two arrays of coloured balls held in vertical tubes. In the planning condition, the participant was required to indicate the minimum possible number of moves needed to rearrange the balls in the upper half of the screen so that they were in the same configuration as those in the lower half of the screen. Participants were instructed that the balls had to be moved in and out of the top of the tubes, could only be moved one at a time and could not be moved past each other in the tubes. In the counting condition, participants had to subtract the number of balls in the top array from the number in the bottom. Once they have identified their response, they reported their answer by pressing the corresponding button on an fMRI compatible button box placed

beneath their right hand. For planning and counting trials, participants were asked to indicate the correct number by selecting from numbers (1-4) presented at the bottom of the screen. For both tasks, problems with correct responses 2, 3, or 4 only were included. More difficult levels of planning were not incorporated in the present experimental paradigm since we were interested in cross-groups differences relating to neural activity associated with planning processes, i.e., rather than differences in overt behavior. Feedback for correct and incorrect answers, consisting of the word “correct” or “incorrect” was provided at the end of each trial. The ‘one-touch’ design ensured that any activity differences across levels of difficulty related to the complexity of planning processes, i.e., rather than the number of overt motor responses. The experimental paradigm lasted for 10 minutes. Planning and counting problems were displayed alternately with an intervening rest interval the duration of which was jittered between 5 and 15 seconds. The difficulty level of the displayed problems varied according to a predefined pseudo-randomized sequence. The duration of problem-solving events was response driven to allow calculation of activation per unit time spent planning; hence, the number of problems completed by each subject varied.

All subjects underwent a training session before scanning to ensure that they understood the rules of the task and were able to perform it adequately. None of the participants were tested on similar tasks in previous experimental sessions (i.e., all the participants were task-naïve).

Comparing total number of planning problems solved separately for 2, 3, and 4 move problems showed a weak effect of Group for the 4 move problems with patients solving fewer problems than controls ($F_{(2,57)}$=3.580, p=0.034; Controls vs. Patients=0.012, Controls vs. Relatives=0.055, values 1-tailed). For the most difficult level of planning (4 moves), patients solved on average 6 problems (sd=1.10), while controls solved on average 6.65 problems (sd=0.49). Because response time is directly related to the number of problems

solved, we accounted for this minimal difference in the number of problems solved by including response times as a covariate in the between group-effects analysis on goal-directed planning.

Image Acquisition

fMRI data were acquired on a 3T Siemens Magnetom Trio scanner. Functional T2*-weighted echoplanar images were acquired parallel to the intercommissural line with the following parameters: TR = 2000 ms, TE = 30 ms, flip angle = 78°; matrix = 64 x 64, FOV = 192 x 192 mm with 32 slices per volume (slice thickness = 3 mm; interslice gap = 25%) giving 3 x 3 mm in-plane resolution. Prior to data analysis, the first 10 images were discarded for T1 equilibration. A structural T1-weighted scan was acquired for co-registration (TR = 2250 ms, TE = 2.99 ms, TI = 900 ms, flip angle = 9°, FOV = 256 x 240 x 192 mm). We did not collect DTI data on this sample.

Imaging Preprocessing

Prior to statistical analysis, imaging data were pre-processed with the Automatic analysis [13] (aa) version 1 batch system using aarecipe_general_ver02.m (http:// imaging.mrccbu.cam. ac.uk/imaging/Automatic Analysis-ManualReference) in SPM8. The steps included in the pre-processing entailed realignment so that for each scanning session, all functional volumes were realigned to the first one in the time series. Data were then slice-time corrected and co-registered with the SPM EPI template. Data were segmented and normalized to the Montreal Neurological Institute (MNI) template and smoothed with an 8mm full width half maximum Gaussian kernel. The data were high-passed filtered (cut-off period = 180 seconds) to remove low frequency drifts in the MRI signal. For two subjects this pipeline did not provide a good normalization so that standard SPM normalization routine was used instead. Imaging data

were analysed using SPM8 (Statistical Parametric Mapping, Wellcome Department of Imaging Neuroscience, London, UK, http://www.fil.ion.ucl.ac.uk/spm). Movement within the scanner was assessed for each participant by calculating the average displacement in each translation and rotational axis. The totals of these averages were then compared across groups. The groups did not differ in movement within the scanner on any of the translation and rotational axis (all $p > 0.347$). The six movement parameters produced during realignment were also included in the first-level models as nuisance variables.

Imaging Analysis

Contrasts

Planning minus counting. In line with previous studies that have used this paradigm [14–16], we used the contrast "planning minus counting" to effectively localize a network that is well known to be involved in spatial planning. The contrast "planning minus counting" across all participants allowed validation of the task with respect with previous studies by identifying a well-recognized network. In addition, it was instrumental to select frontal ROI for the PPI analysis for between-groups comparisons by crucially avoiding the potential confound of 'double dipping'. This contrast localized a network involved in spatial planning, but such activation is not "selective" for planning and can be identified in relation to other complex cognitive tasks (i.e. relational reasoning), even via data driven methods such as Independent Component Analysis [17]. After specifying the contrast of interest for each individual, whole-brain maps were collated for second-level (group) random-effect analyses. The main effect of planning was therefore identified by performing a one-sample t-test of "planning minus counting" events across all participants.

Planning minus resting, between-group differences. In line with previous studies that have used this paradigm [14], planning activation relative to rest for 2, 3, and 4 moves was used to address between-group differences. The contrast “planning minus resting” is characterized by more reliable signal and therefore greater sensitivity for detecting cross-group effects. In fact sensitivity to brain dysfunction has been previously identified using this contrast in patients affected by skin picking disorder [15] and National Football League players with executive deficits [14]. This contrast comprehensively detected all the cognitive processes involved during goal-directed planning, including processes of visuospatial perception and attention. Accordingly, between-group differences during planning were analysed using a 3x3 full factorial model, with planning complexity as within-subject factor, (planning activation relative to rest for 2, 3, and 4 move problems) and group as the between-subject factor (controls, relatives, and patients). In addition, we analysed whole-brain between-group differences via a One-Way ANOVA on the contrast “planning minus resting”.

“Planning minus resting” in combination with ROI analysis also revealed caudate involvement (which was not detected on the contrast “planning minus counting” consistently with several studies addressing planning activation vs. control condition [6,7,14,16]). Striatal involvement has been previously associated specifically with planning difficulty [7]. Therefore, even if we did not include higher level of complexity in the task, we reasoned that “planning vs. resting” was more sensitive in order to capture caudate activation possibly modulated by planning complexity (see also below planning high – planning low contrast).

Planning minus counting, between-group differences. To identify between-group difference in the cognitive processes selectively involved during goal-directed planning we used a 3x3 full factorial model with planning complexity relative to counting for 2, 4, and 4 moves as within-subject factor and group as the between-subject factor (controls, relative, and

patients). Accordingly, for each individual the contrasts "planning 2 minus counting 2", "planning 3 minus counting 3", planning 4 minus counting 4" were estimated and whole-brain maps depicting this contrast collated for second-level (group) random-effects analyses.

Planning high minus planning low. We used the contrast "planning high minus planning low" to assess brain modulation in response to planning difficulty. Accordingly, for each individual the contrast "planning 4 minus planning 2" was estimated and whole-brain maps depicting this contrast collated for second-level (group) random-effects analyses. Between-groups differences were assessed via a One-way ANOVA and activation was deemed significant at p<0.05, family-wise error (FWE) corrected at the voxel level.

Counting minus resting. To rule out that between-group differences in brain were not driven by abnormality of processes involved in performing counting trials, we used a 3x3 full factorial model, with counting complexity as within-subject factor, (counting activation relative to rest for 2, 3, and 4 move problems) and group as the between-subject factor (controls, relatives, and patients). Similarly to what reported in the main text for the main contrast "planning minus resting", activation was deemed significant at p <0.05, family-wise error (FWE) corrected at the voxel level. In addition, we analysed whole-brain between-groups differences via a One-Way ANOVA on the contrast "counting vs. resting".

Regions of interest

Although we did not observe significant striatal activation in association with the planning component of the Tower Of London (TOL), caudate and putamen are significantly involved in OCD pathophysiology [18] and may be relevant in terms of mediating executive performance in OCD [19,20] via their connections with the prefrontal cortex. Previous

studies have demonstrated caudate activation during TOL in association with task difficulty (see also Contrasts section). Small regions of interest were specified to conduct analyses involving subcortical structure (i.e., caudate and putamen). Relying on ROI based the Automated Anatomical Labeling (AAL) implemented with SPM would have resulted in the selection of the all caudate and putamen with a significant lack of specificity and averaging over different functional territories. Therefore, in line with previous work [20], we used coordinates based on AFNI-supplied atlas (TT-Daemon atlas) and appropriately transformed to MNI coordinates, on which we built small spherical ROIs on 3.5mm diameter.

We have previously reported that resting state connectivity between the right putamen (ROI at x=24, y=0, z=3) and the right DLPFC was specifically related to planning performance in OCD patients [20]. Therefore, the peak coordinates for the putamen were at MNI coordinates x=24, y=0, z=3. Of the AFNI-supplied atlas coordinates, coordinates for the caudate at MNI x=11, y=7, z=9 corresponded to the body of the caudate and were located in the functional territory of the frontoparietal control network identified by Choi and colleagues [21] which is likely involved in the executive function processes investigated in this study.

Functional connectivity

For each participant, the time-course was extracted from a 10 mm radius spherical ROI using the Volume of Interest function, extracting the first eigenvector from all voxels within the ROI. The extracted time-course was deconvolved to estimate underlying neural activation and multiplied point wise with a psychological function in which time points during planning are designated as 1, during rest as 0 and during counting as -1. The resultant psychological, physiological and PPI time-courses were convolved with the hemodynamic response function

to generate a predictor of BOLD activation and entered into a general linear model along with the six movement parameters included as effects of no interest.

For target caudate and putamen *a priori* defined ROIs, the beta values for the PPI, indexing FC during planning, were extracted and imported in SPSS. Similarly, once activation due to the specified psychological conditions had been accounted for, beta values for the physiological predictor indexing general connectivity were examined.

Separately for the PPI and the physiological predictor, data were collapsed within groups and examined via one-sample t-test. Inspection of PPI between the frontal and the putamen seed, revealed the presence of two outliers (> 2 standard deviations from the group mean), which were removed from primary analysis, but the inclusion or exclusion of which did not affect the main findings. PPI significant effects were tested across all participants. In the case of significant modulation (i.e., significant connectivity), between group differences in PPI FC were computed. For between-group comparisons, the default Monte Carlo procedure implemented in SPSS was used. In the case in which the sample size is small and the asymptotic method unsuitable, the Monte Carlo procedure represents a valid alternative. The Monte Carlo method is a repeated sampling method, which estimates p values by taking a random sample from a reference set. We used the default number of samples used in calculating Monte Carlo approximation, namely 10,000 with a random seed generation and default value 99 for the confidence level. The Monte Carlo algorithms implemented in SPSS make use of ideas from several papers [22–25] and description of the procedure can be found in Mehta and Patel [26].

Before examining between-group differences in frontostriatal connectivity, we explored the beta parameter estimated of task-related activation for the 10 mm sphere built in the DLPFC (x=24, y=20, z=52) for the contrast “planning vs. counting”. Significant activation was found in controls (t_{18}=5.291, p<0.001), relatives (t_{17}=3.037, p=0.007), and

patients (t_{20}=3.693, p=0.001). Therefore, as significant activation was found in each group, we confidently examined the beta weights of the PPI predictor for the contrast “planning vs. counting” for comparison between groups.

SUPPLEMENTAL RESULTS

Imaging Results

Planning minus counting. As reported in the main text a robust effect of “planning minus counting” was found in the expected dorsal fronto-parietal network across all participants ($p<0.05$, FWE). Isolation of this network is highly consistent with previous findings using the same experimental paradigm [14–16]. Residual activation in the precuneus and lingual gyrus might suggest differential visuo-motor demands on counting and planning trials. However, motor demands were carefully balanced by the one touch design and these brain regions are often active in tasks that do not have overt motor demands [14].

Planning minus resting. ROI analysis was conducted for the right putamen and right caudate for the contrast “planning minus resting”. Parameter estimate signal change was explored separately in each group by means of one-sample t-test. Significant caudate activation was found in controls (t_{19}=5.759, $p<0.001$), relatives (t_{18}=3.194, p=0.005), and patients (t_{20}=5.578, $p<0.001$). To test whether the caudate was significantly more active than the putamen in the contrast “planning vs. resting”, contrast estimates for these ROIs were entered in a repeated measures analysis of variance. The caudate was significantly more active than the putamen ($F_{1,57}$=66.244, $p<0.001$) with no main effect of group (p=0.502) or ROI by group interaction (p=0.321).

Effect of task load on brain activation. We investigated brain modulation in relation to the contrast "planning high minus planning low" in order to assess brain modulation in response to planning difficulty. In line with previous findings [7], we replicated activation of subcortical brain structures for increased planning difficulty in controls with activation of the thalamus (x=12, y=-4, z=4, $p<0.05$, FWE). There was no such modulation in relatives nor in patients with OCD. At lower level of statistical threshold ($p<0.001$, uncorrected), parietal (x=-2, y=-60, z=52) and caudate (x=8, y=4, z=16) activation was also identified. There was no a main effect of group on this contrast as assessed via a One-way ANOVA and there was no evidence of increased activation in patients compared with controls at $p<0.05$ FWE nor at lower level of statistical correction ($p<0.001$, uncorrected).

Between-group effects on counting. There were no between group differences for counting trials at $p<0.05$, FWE corrected nor for more lenient level of statistical correction (0.001, uncorrected).

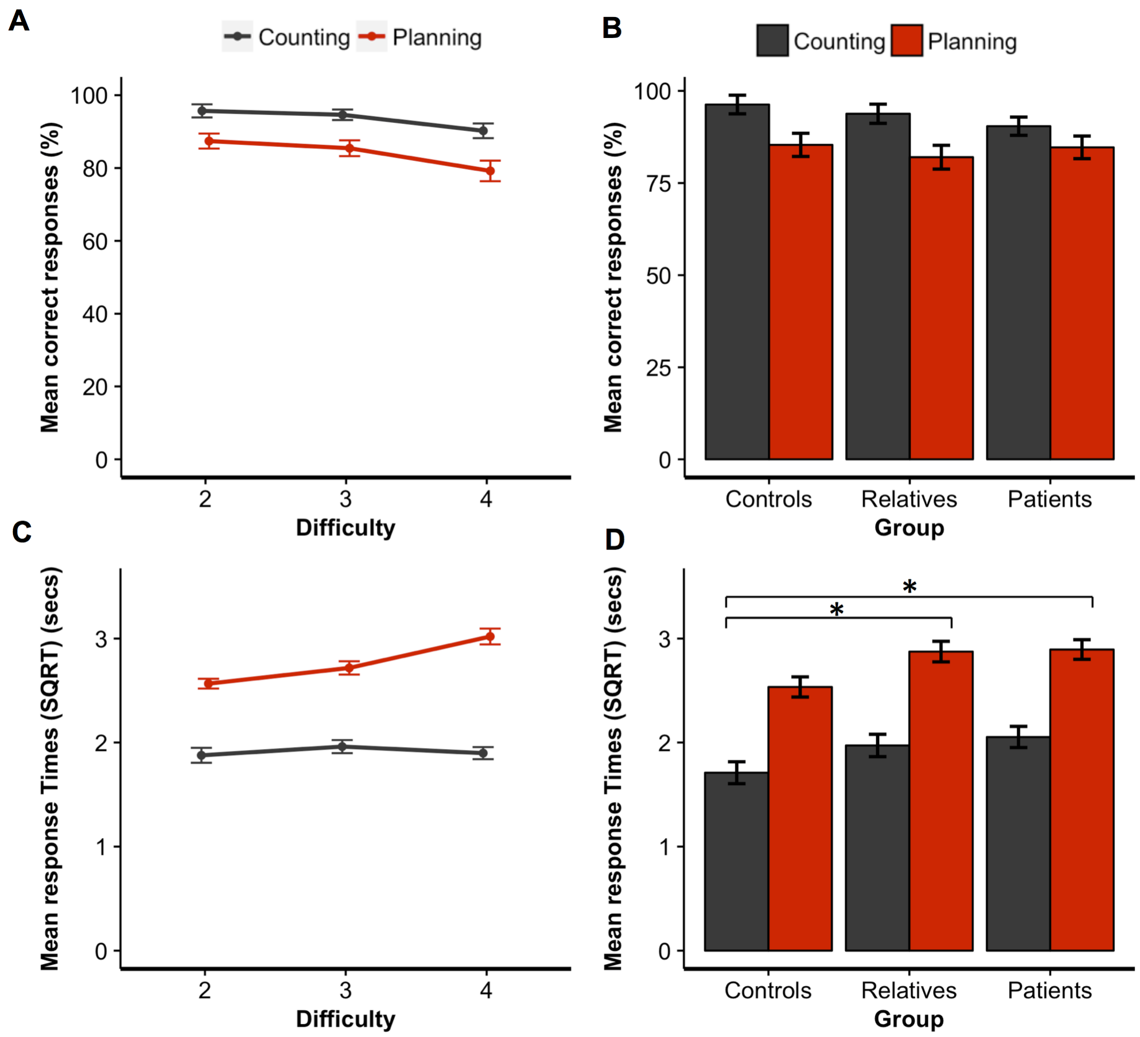

Figure S1. Behavioural Measures Related to Counting and Planning in OCD Patients, Their First-Degree Unaffected Relatives and Control Subjects

(A) Mean accuracy for different levels of difficulty (2, 3 or 4 moves), revealing a main effect of task and a main effect of difficulty. There was not a significant interaction between task and difficulty (see main text). **(B)** There was no significant difference between groups in accuracy of performance (see main text). **(C)** Mean response times for different levels of difficulty revealing a main effect of task, a main effect of difficulty, and a significant task by difficulty interaction (see main text). **(D)** There was a main effect of group on response times, with patients and relatives being slower than controls irrespective of task and difficulty level (see main text). Error bars denote standard error mean (SEM).

Table S1. Brain Areas Activated During Planning Relative to Counting Collapsed Across All Participants (n=60)

	Region	BA	MNI Coordinates x y z	k_E	Z	Peak p_{FWE}
L	Precuneus	7	-4 -56 48	2929	Inf	<0.001
R	Precuneus		4 -56 46		Inf	<0.001
R	Precuneus		6 -62 24		5.94	<0.001
R	Angular Gyrus	39/40	44 -68 32	2445	7.75	<0.001
R	Angular Gyrus		50 -50 36		7.32	<0.001
L	Middle Temporal Gyrus	39	-40 -74 28	1884	6.86	<0.001
L	Inferior Parietal Lobule		-48 -54 42		5.85	<0.001
L	Middle Temporal Gyrus		-26 -58 22		4.48	0.021
R	Superior Frontal Gyrus	8/6	24 20 52	1220	6.21	<0.001
R	Middle Frontal Gyrus	8/9	32 24 46		6.03	<0.001
R	Middle Frontal Gyrus	6/8	38 12 50		5.16	0.001
L	Cingulate cortex		-20 2 30	172	4.81	0.005
L	Cingulate Gyrus		-18 -10 34		4.75	0.007
L	Middle Temporal Gyrus		-36 -54 2	13	4.58	0.014
L	Lingual Gyrus	18/30/17	-4 -78 -2	70	4.56	0.015
L	Lingual Gyrus		-12 -72 -6		4.42	0.026
L	Inferior Parietal Lobule		-28 -38 30	35	4.56	0.015
L	Middle Frontal Gyrus	44/45	-44 22 38	39	4.56	0.015
L	Superior Frontal Gyrus	6/8	-34 12 54	30	4.54	0.016
L	Cerebellum/Lobule VI, VIIa Crus I		-10 -82 -24	12	4.51	0.018
L	Middle Frontal Gyrus	9	-28 20 30	2	4.3	0.041
R	Superior Medial Gyrus	6/8/9	4 36 42	3	4.25	0.048
L	Cingulate Gyrus	31	-24 -26 34	1	4.25	0.048

Coordinates in MNI space. p_{FWE} = p value with family-wise error correction for the whole brain volume ($p< 0.05$). R, right; L, left; BA, Brodmann Area; MNI, Montreal Neurological Institute; k_E, cluster size; Z, Z score; FWE, family-wise error.

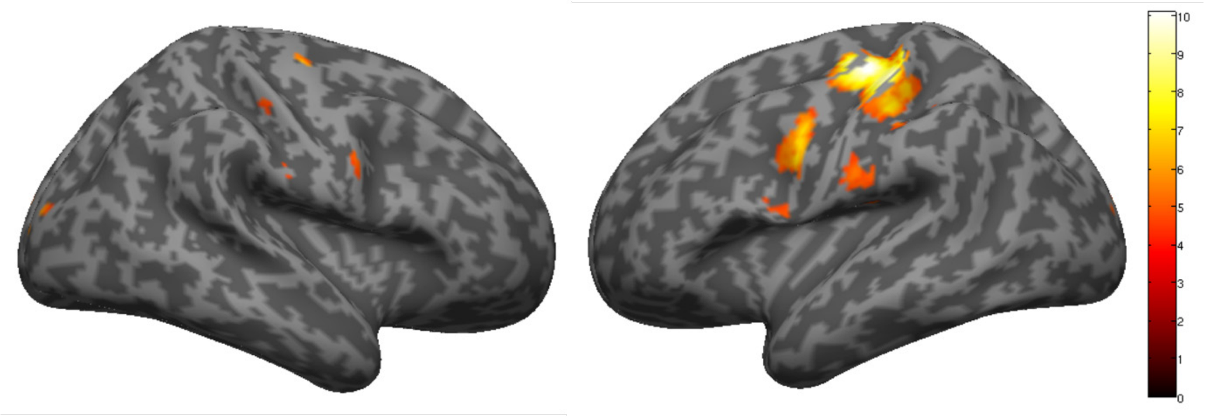

Figure S2. Brain Network for Counting vs. Planning Across All Participants (n=60)

Voxel-wise FWE correction for the whole brain mass p < 0.05.

Table S2. Brain Areas Activated During Counting Relative to Planning Collapsed Across All Participants (n=60)

Region		BA	MNI Coordinates x y z	k_E	Z	Peak p_{FWE}
L	Precentral Gyrus	4	-36 -22 58	1310	7.67	<0.001
R	Medial Frontal Gyrus (SMA)	6	6 2 54	1263	6.52	<0.001
L	Medial Frontal Gyrus	6	-4 0 54		6.46	<0.001
R	Putamen		24 8 0	334	6.11	<0.001
L	Precentral Gyrus	6	-56 4 30	460	6.08	<0.001
L	Inferior Frontal Gyrus	44	-52 6 8		4.49	0.02
L	Putamen		-24 2 -2	444	5.95	<0.001
R	Precentral Gyrus	6	36 -14 60	54	5.61	<0.001
R	Superior Occipital Gyrus	18	20 -94 16	66	5.48	<0.001
R	Cerebellum (VI)		22 -52 -22	208	5.33	<0.001
L	Thalamus		-12 -20 6	106	5.18	0.001
R	Precentral Gyrus	6	58 4 36	51	4.99	0.002
L	Postcentral Gyrus	3	-58 -20 24	136	4.97	0.003
L	Insula	13	-46 -24 22		4.83	0.005
L	Superior Occipital Gyrus	18	-16 -96 18	26	4.93	0.003
R	Postcentral Gyrus	2	48 -26 46	23	4.59	0.013
R	Supramarginal Gyrus	3	60 -16 24	5	4.33	0.036
L	Insula	13	-36 -2 12	1	4.33	0.037

Coordinates in MNI space. BA, Brodmann Area; p_{FWE} = p value with family-wise error correction for the whole brain mass. R, right; L, left; MNI, Montreal Neurological Institute; k $_E$, cluster size; Z, Z score; FWE, family-wise error.

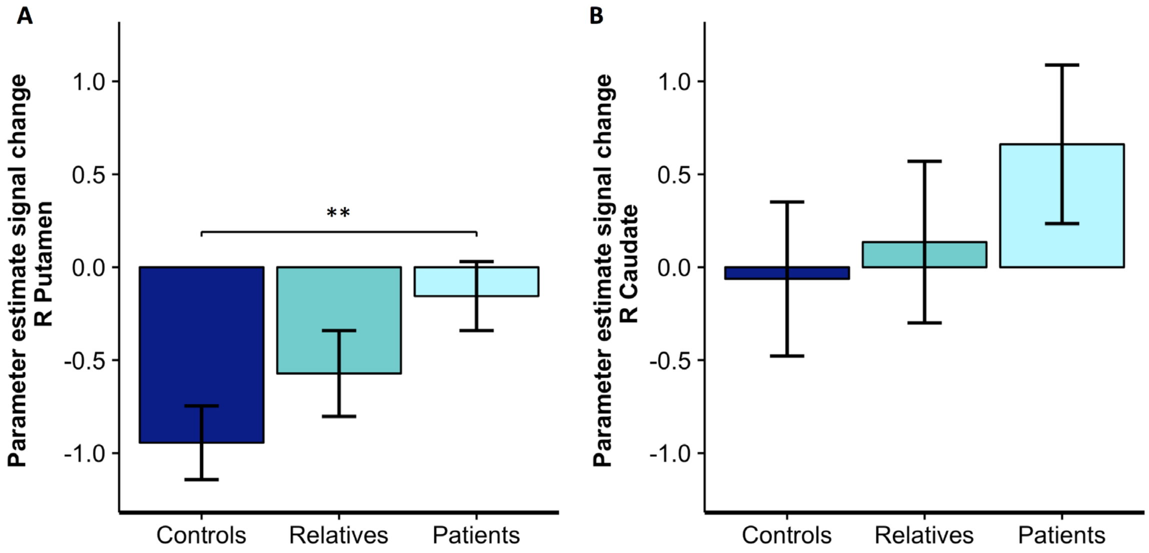

Figure S3. ROI Analysis for the Contrast Planning Minus Counting

ROI analysis for the contrast planning minus counting in right putamen (A) and right caudate (B). Error bars denote standard error mean (SEM). R, right. **p=0.007.

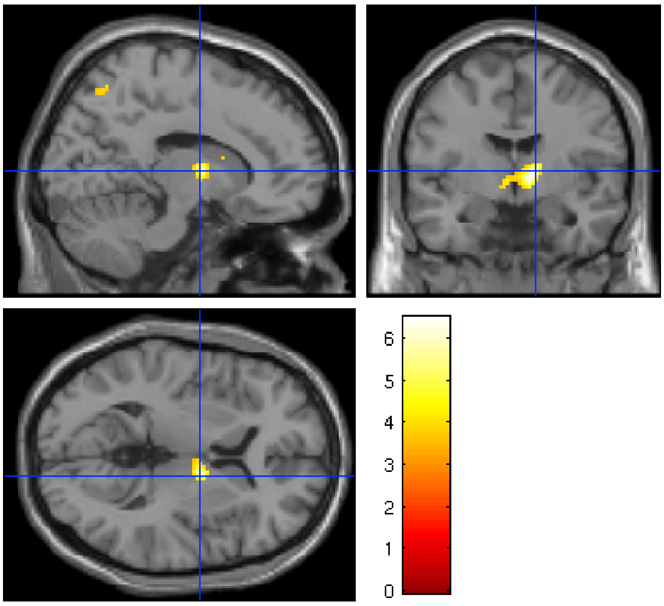

Figure S4. Brain Activation in Control Participants for High vs. Low Planning Difficulty

Voxel-wise p<0.001 uncorrected.

SUPPLEMENTAL REFERENCES

1. Goodman WK, Price LH, Rasmussen SA, Mazure C, Fleischmann RL, Hill CL, et al. The Yale-Brown Obsessive Compulsive Scale. I. Development, use, and reliability. Arch. Gen. Psychiatry. 1989;46:1006–11.

2. Oldfield RC. The assessment and analysis of handedness: the Edinburgh inventory. Neuropsychologia. 1971;9:97–113.

3. Chamberlain SR, Menzies L, Hampshire A, Suckling J, Fineberg NA, del Campo N, et al. Orbitofrontal dysfunction in patients with obsessive-compulsive disorder and their unaffected relatives. Science. 2008;321:421–2.

4. Shallice T. Specific impairments of planning. Philos. Trans. R. Soc. Lond. B. Biol. Sci. 1982;298:199–209.

5. Baker SC, Rogers RD, Owen AM, Frith CD, Dolan RJ, Frackowiak RS, et al. Neural systems engaged by planning: a pet study of the tower of london task. Neuropsychologia. 1996;34:515–26.

6. Owen AM, Doyon J, Petrides M, Evans AC. Planning and spatial working memory: a positron emission tomography study in humans. Eur. J. Neurosci. 1996;8:353–64.

7. Dagher A, Owen AM, Boecker H, Brooks DJ. Mapping the network for planning: a correlational PET activation study with the Tower of London task. Brain J. Neurol. 1999;122 (Pt 10):1973–87.

8. Schall U, Johnston P, Lagopoulos J, Jüptner M, Jentzen W, Thienel R, et al. Functional brain maps of Tower of London performance: a positron emission tomography and functional magnetic resonance imaging study. NeuroImage. 2003;20:1154–61.

9. Lazeron RHC, Rombouts SARB, Machielsen WCM, Scheltens P, Witter MP, Uylings HBM, et al. Visualizing brain activation during planning: the tower of london test adapted for functional mr imaging. Am. J. Neuroradiol. 2000;21:1407–14.

10. Newman SD, Carpenter PA, Varma S, Just MA. Frontal and parietal participation in problem solving in the Tower of London: fMRI and computational modeling of planning and high-level perception. Neuropsychologia. 2003;41:1668–82.

11. van den Heuvel OA, Groenewegen HJ, Barkhof F, Lazeron RHC, van Dyck R, Veltman DJ. Frontostriatal system in planning complexity: a parametric functional magnetic resonance version of tower of london task. NeuroImage. 2003;18:367–74.

12. van den Heuvel OA, Veltman DJ, Groenewegen HJ, Cath DC, van Balkom AJLM, van Hartskamp J, et al. Frontal-striatal dysfunction during planning in obsessive-compulsive disorder. Arch. Gen. Psychiatry. 2005;62:301–9.

13. Cusack R, Vicente-Grabovetsky A, Mitchell DJ, Wild CJ, Auer T, Linke AC, et al. Automatic analysis (aa): efficient neuroimaging workflows and parallel processing using Matlab and XML. Front. Neuroinformatics. 2014;8:90.

14. Hampshire A, MacDonald A, Owen AM. Hypoconnectivity and hyperfrontality in retired American football players. Sci. Rep. 2013;3:2972.

15. Odlaug BL, Hampshire A, Chamberlain SR, Grant JE. Abnormal brain activation in excoriation (skin-picking) disorder: evidence from an executive planning fMRI study. Br. J. Psychiatry. 2016;208:168–74.

16. Williams-Gray CH, Hampshire A, Robbins TW, Owen AM, Barker RA. Catechol O-Methyltransferase val158met Genotype Influences Frontoparietal Activity during Planning in Patients with Parkinson's Disease. J. Neurosci. 2007;27:4832–8.

17. Parkin BL, Hellyer PJ, Leech R, Hampshire A. Dynamic Network Mechanisms of Relational Integration. J. Neurosci. 2015;35:7660–73.

18. Graybiel AM, Rauch SL. Toward a neurobiology of obsessive-compulsive disorder. Neuron. 2000;28:343–7.

19. Alexander GE, Crutcher MD. Functional architecture of basal ganglia circuits: neural substrates of parallel processing. Trends Neurosci. 1990;13:266–71.

20. Vaghi MM, Vértes PE, Kitzbichler MG, Apergis-Schoute AM, van der Flier FE, Fineberg NA, et al. Specific Frontostriatal Circuits for Impaired Cognitive Flexibility and Goal-Directed Planning in Obsessive-Compulsive Disorder: Evidence From Resting-State Functional Connectivity. Biol. Psychiatry. 2017;81:708–17.

21. Choi EY, Yeo BTT, Buckner RL. The organization of the human striatum estimated by intrinsic functional connectivity. J. Neurophysiol. 2012;108:2242–63.

22. Agresti A, Wackerly D, Boyett J. Exact conditional tests for cross-classifications: Approximation of attained significance levels. Psychometrika. 1979;44:75–83.

23. Patefield WM. Algorithm AS 159: An Efficient Method of Generating Random R × C Tables with Given Row and Column Totals. J. R. Stat. Soc. Ser. C Appl. Stat. 1981;30:91–7.

24. Mehta CR, Patel NR, Senchaudhuri P. Importance Sampling for Estimating Exact Probabilities in Permutational Inference. J. Am. Stat. Assoc. 1988;83:999–1005.

25. Senchaudhuri P, Mehta CR, Patel NR. Estimating Exact p Values by the Method of Control Variates or Monte Carlo Rescue. J. Am. Stat. Assoc. 1995;90:640–8.

26. Mehta, C. R., & Patel, N. R. (2010). IBM SPSS Exact Tests. Somers, NY: SPSS
